# Characterization of hepatitis C RNA-containing particles from human liver by density and size

**DOI:** 10.1099/vir.0.2008/000083-0

**Published:** 2008-10

**Authors:** Søren U. Nielsen, Margaret F. Bassendine, Caroline Martin, Daniel Lowther, Paul J. Purcell, Barnabas J. King, Dermot Neely, Geoffrey L. Toms

**Affiliations:** 1 Liver Research Group, Clinical Medical Sciences, Newcastle University, Newcastle upon Tyne NE2 4HH, UK; 2 Department of Clinical Biochemistry, Royal Victoria Infirmary, Newcastle upon Tyne, UK

## Abstract

Hepatitis C virus (HCV) particles found *in vivo* are heterogeneous in density and size, but their detailed characterization has been restricted by the low titre of HCV in human serum. Previously, our group has found that HCV circulates in blood in association with very-low-density lipoprotein (VLDL). Our aim in this study was to characterize HCV RNA-containing membranes and particles in human liver by both density and size and to identify the subcellular compartment(s) where the association with VLDL occurs. HCV was purified by density using iodixanol gradients and by size using gel filtration. Both positive-strand HCV RNA (present in virus particles) and negative-strand HCV RNA (an intermediate in virus replication) were found with densities below 1.08 g ml^−1^. Viral structural and non-structural proteins, host proteins ApoB, ApoE and caveolin-2, as well as cholesterol, triglyceride and phospholipids were also detected in these low density fractions. After fractionation by size with Superose gel filtration, HCV RNA and viral proteins co-fractionated with endoplasmic reticulum proteins and VLDL. Fractionation on Toyopearl, which separates particles with diameters up to 200 nm, showed that 78 % of HCV RNA from liver was >100 nm in size, with a positive-/negative-strand ratio of 6 : 1. Also, 8 % of HCV RNA was found in particles with diameters between 40 nm and 70 nm and a positive-/negative-strand ratio of 45 : 1. This HCV was associated with ApoB, ApoE and viral glycoprotein E2, similar to viral particles circulating in serum. Our results indicate that the association between HCV and VLDL occurs in the liver.

## INTRODUCTION

*Hepatitis C virus* (HCV) belongs to the family *Flaviviridae* and is infectious in humans and chimpanzees (Lindenbach & Rice, 2001). There are some biochemical and biophysical data for HCV virus particles from infected hosts (André *et al.*, 2002; Petit *et al.*, 2005; Roingeard *et al.*, 2004) but the characterization of native virus particles has been difficult due to the low titre of HCV in serum. Analysis of HCV from human serum by immunoprecipitation and Western blotting shows that the virus particle contains glycoproteins E1 and E2 (Petit *et al.*, 2005), and that virus particles are associated with apolipoprotein B and E (ApoB and ApoE) (André *et al.*, 2002; Nielsen *et al.*, 2006). A more detailed structural analysis of native HCV particles from infected hosts requires higher titres of virus. However, the characterization of HCV produced *in vitro* has been facilitated by two scientific breakthroughs.

The first breakthrough in the characterization of HCV produced *in vitro* was the development of the replicon system (Lohmann *et al.*, 1999). In this system, HCV (genotypes 1b and 2a) replicates in Huh-7 human hepatoma cells and produces all HCV structural and non-structural (NS) proteins, as well as positive- and negative-strand HCV RNA (Lohmann *et al.*, 1999; Pietschmann *et al.*, 2002; Quinkert *et al.*, 2005). Although virus particles are not secreted from Huh-7 cells transfected with the replicon, the system has enabled detailed studies of subcellular structures involved in HCV replication and characterization of the HCV replication complex. The replication complex is found inside a membranous web which also contains lipid rafts (Gosert *et al.*, 2003; Shi *et al.*, 2003) and has a low density (≤1.10 g ml^−1^) (El-Hage & Luo, 2003). More than 30 % of proteins associated with partially purified replication complexes are involved in lipid metabolism (Huang *et al.*, 2007). Further evidence for the role of host lipid metabolism in HCV replication comes from Kapadia & Chisari (2005). This study demonstrated that HCV RNA replication is increased in Huh-7 cells when the growth medium is supplemented with monounsaturated fatty acids, but reduced when cells are grown in the presence of polyunsaturated fatty acids.

The second breakthrough in the characterization of HCV produced *in vitro* was the identification of the JFH-1 strain (Kato *et al.*, 2001). This genotype 2a virus replicates in cell culture in Huh-7 and Huh-7.5 cells to produce infectious virus (Lindenbach *et al.*, 2005; Wakita *et al.*, 2005). The buoyant densities of virus produced from the JFH-1 strain cover a wide range between 1.03 g ml^−1^ and 1.16 g ml^−1^. The peak of HCV RNA coincides with fractions of lowest infectivity that have a density around 1.14 g ml^−1^, a sedimentation coefficient of 200 S and a diameter of approximately 55–70 nm (Gastaminza *et al.*, 2006; Wakita *et al.*, 2005). The buoyant density of infectious virus produced *in vitro* by the JFH-1 strain is below 1.10 g ml^−1^ (Chang *et al.*, 2007; Gastaminza *et al.*, 2006). These biophysical properties are similar to HCV obtained from the blood of infected patients, where HCV has been shown to be associated with very-low-density lipoprotein (VLDL), a lipid particle that contains triglyceride, phospholipid, ApoB and ApoE (André *et al.*, 2002; Nielsen *et al.*, 2006; Prince *et al.*, 1996).

The proportion of HCV circulating in blood which has low density and is associated with VLDL varies between patients (Kanto *et al.*, 1995; Zahn & Allain, 2005). This paper aims to determine the density and size of HCV from human liver and suggest the subcellular location in which HCV becomes associated with VLDL, information that is currently unknown.

We have previously demonstrated the presence of viral structural proteins in the explant liver of a patient with HCV infection and common variable immunodeficiency by Western blotting and immunohistochemistry (Fenwick *et al.*, 2006; Nielsen *et al.*, 2004). Our present work characterizes the HCV RNA-containing particles in this patient's liver by density and size and provides new insight into the biochemical composition and assembly of HCV in human liver.

## METHODS

### HCV samples from patients.

Patient S6 suffered from common variable immunodeficiency and was infected with the 1a genotype of HCV from contaminated intravenous immunoglobulin (Christie *et al.*, 1997). The viral infection progressed rapidly to cirrhosis, requiring a liver transplant. However, the transplant liver (S6b) failed and was removed at retransplant; it was found to contain a high titre of HCV (5×10^9^ IU per gram liver) (Pumeechockchai *et al.*, 2002). An HCV-negative liver sample was obtained from a patient with a Klatskin tumour in the hepatic bile ducts. Cubes of 1 g either HCV-infected liver or control HCV-negative liver were immersed in ice-cold homogenization buffer (Nielsen *et al.*, 2004) and a macerate was produced using a tight-fitting Dounce homogenizer (VWR). The collection of human serum and tissue samples was made with informed consent and the research project was approved by the Joint Ethics Committee of the Newcastle and North Tyneside Health Authority, Newcastle University and University of Northumbria.

### Iodixanol density gradients.

Preformed iodixanol (Optiprep; Axis-Shield) density gradients were prepared from two buffered solutions of iodixanol at 6 % (1.7 ml 60 %, w/v, iodixanol, 0.34 ml 0.5 M Tris/HCl, pH 8.0, 0.34 ml 0.1 M EDTA, pH 8.0 and 14.6 ml 0.25 M sucrose) and 56.4 % (16.0 ml 60 %, w/v, iodixanol, 0.34 ml 0.5 M Tris/HCl, pH 8.0, 0.34 ml 0.1 M EDTA, pH 8.0 and 0.34 ml 0.25 M sucrose). The gradient was harvested from the bottom by tube puncture using a model 184 tube piercer (Isco) and collected in 14 fractions. The density of each fraction was determined using a digital refractometer (Atago). Self-forming iodixanol gradients (Graham *et al.*, 1994) were prepared by adding 0.2 ml 0.5 M Tris/HCl (pH 8.0), 0.34 ml 0.5 M EDTA, 4.2 ml 60 % (w/v) iodixanol and 1.5 ml 0.25 M sucrose into thick-walled Ti-50 polycarbonate centrifuge tubes (Beckman). Each centrifuge tube then received 4.2 ml liver macerate and the content was thoroughly mixed to form a homogeneous solution. These gradients were centrifuged at 50 000 r.p.m. in a Beckman L8-70M ultracentrifuge using a model Ti-50 rotor for 24 h at 4 °C and harvested manually from the top, collecting 18 fractions of 0.5 ml each from each sample.

For electron microscopy (EM) analysis, iodixanol fractions were dialysed against 100 mM Tris/HCl, pH 7.4, with 2 mM EDTA at 4 °C. Samples for thin sectioning were fixed with 3 % glutaraldehyde (EM grade; VWR) and embedded in LR White (London Resin). Samples for negative staining were applied to Formvar-coated and glow-discharged copper grids; these were washed with Sorenson's buffer and stained with 1 % uranyl acetate. Grids were viewed on a Philips CM 100 transmission electron microscope equipped with an AMT CCD camera.

### Quantification of HCV RNA by real-time RT-PCR.

Real-time RT-PCR (qRT-PCR) for positive-strand HCV RNA was carried out as described previously (Mercier *et al.*, 1999; Nielsen *et al.*, 2004) using primers NCR-3 and NCR-5 (Mercier *et al.*, 1999) plus a fluorescent probe (5′-FAM-ATTCCGGTGTACTCACCGGTTCCGCAGA-TAMRA-3′). Primers NCR-3 and -5 anneal between nucleotides 120 and 290 in the 5′ non-translated region of the HCV 1a genome. The HCV positive-strand assay was calibrated against WHO international standard for HCV 96/790 from the National Institute of Biological Standards and Controls. Negative-strand HCV RNA was detected using a qRT-PCR assay with the tagged primer, NCR-9 (Komurian-Pradel *et al.*, 2004; Nielsen *et al.*, 2006). NCR-9 (5′-GCGTCGGCAGTATCGTGAATTCGACCCCCCCTCCCGGGAGAGCCAT-3′; the tag is underlined) anneals to the 3′ non-translated region of the negative strand and was used for reverse transcription. Residual RNA template was removed with RNase A and RNase H (GE Healthcare). The cDNA was quantified by qRT-PCR using primer NCR-8 (5′-CGTCGGCAGTATCGTGAATTC-3′), which anneals to the tag sequence of NCR-9, in combination with NCR-3 and the fluorescent probe. To calibrate this assay, synthetic negative-strand HCV RNA was prepared by *in vitro* transcription using a T7 Megascript kit (Ambion). A 702 bp DNA fragment between nucleotides 1 and 702 of the HCV RNA genome was cloned by RT-PCR using a forward (5′-CGCGGATCCCCCCTGTGAGGAACTACTGTCTTCAC-3′; the *Bam*HI restriction site is underlined) and a reverse (5′-CGCAAGCTTGCACGTAAGGGTATCGATGACCTTAC-3′; the *Hin*dIII restriction site is underlined) primer. The *Bam*HI–*Hin*dIII-restricted DNA fragment was subcloned into pBluescript (+) (Stratagene). Negative-strand HCV RNA was synthesized from the linerized plasmid by *in vitro* transcription and was purified by acrylamide/urea RNA gel electrophoresis. The band of negative-strand HCV RNA was eluted with SDS and the copy number was calculated from the *A*_260_ (NanoDrop).

### SDS-PAGE and matrix-assisted laser desorption/ionization–time of flight mass spectrometry (MALDI-TOF MS) analysis.

Proteins in iodixanol fractions were analysed on SDS-polyacrylamide gradient gels (3–18 %). Proteins were stained with Coomassie brilliant blue G-250 (Sigma) and excised from the gel for MALDI-TOF MS. Peptides generated by trypsin digestion were analysed using a Voyager DE-STR mass spectrometer (Applied Biosystems). Protein bands were identified by performing searches using the peptide mass fingerprint data and the Mascot search engine program (Matrix Science), searched against the latest NCBI protein sequence database. Only proteins with Mascot scores above 64, which shows that the likelihood of a correct match is significant (*P*<0.05), were accepted as hits.

The monoclonal antibodies (mAbs) used in Western blotting were human anti-HCV E1 glycoprotein (1C4; hybridoma clone IGH398 from Dr A. Union, Innogenetics, Belgium), mouse anti-HCV E2 glycoprotein (AP33; from Dr A. Patel, MRC Virology unit, Glasgow, UK), human anti-HCV core protein (B12; from Professor M. Mondelli, University of Pavia, Italy), mouse anti-HCV NS3 protein (MMM33; LabVision) and human anti-HCV NS4A (D10; from M. Mondelli). The polyclonal antibodies used in Western blotting were rabbit anti-HCV NS5A (from Professor P. Mavromara, Hellenic Pasteur Institute, Athens, Greece) and rabbit anti-human ApoE (DakoCytomation). Western blots were developed using ECL Plus (GE Healthcare) and bands were semi-quantified using a GS-800-calibrated densitometer with Quantity One software (Bio-Rad).

### Quantification of lipids.

Lipids in iodixanol fractions were extracted with chloroform/methanol (Folch *et al.*, 1957). Each 0.5 ml fraction was mixed with 10 ml chloroform/methanol (40 : 60, v/v). Extraction of lipids was performed by rotation at 11 r.p.m. for 2 h at 37 °C followed by centrifugation at 1 000 ***g*** for 5 min. The supernatant was harvested and mixed with 1 ml 100 mM sodium phosphate, pH 7.4. After centrifugation at 2 000 ***g*** for 10 min, the lower, organic phase was evaporated to dryness with nitrogen. The pellet was resuspended in 100 μl 10 mM sodium phosphate, pH 8.0, containing 4 % NP-40 (Roche). Lipids were measured with a Cobas Fara auto analyser (Roche) using phospholipid assay B and free cholesterol E kit (Wako). Triglyceride and total cholesterol were measured using kits from Horiba ABX.

### Gel filtration of lipoproteins and HCV.

Superose 6 prep grade was packed into one XK 16/100 column and one XK 16/40 column (GE Healthcare) and the two columns were run in series. The elution buffer contained 20 mM Tris/HCl (pH 8.0), 0.25 M sucrose, 2 mM EDTA, 2 mM MgSO_4_, 2 mM MgCl_2_ and 0.02 % NaN_3_. The Superose column was calibrated using VLDL, low-density lipoprotein (LDL), and high-density lipoprotein (HDL) purified from normal human plasma (Mackness & Durrington, 1992; März *et al.*, 1993). Toyopearl HW-75S (Tosoh Corporation) was packed into one XK 26/100 column. Sample (2 ml) was applied to the column; the columns were cooled to 4 °C and run with a flow rate of 1 ml min^−1^. Calibration of the Toyopearl column was performed using purified chylomicrons, VLDL, LDL and HDL as well as carboxylated latex bead standards (Okazaki *et al.*, 2005; Magsphere).

A standard curve was prepared by plotting √(−log *K*_AV_) against the bead diameter in nm (Anonymous, 2002), calculated using the following equation:
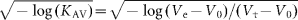
*V*_e_ is the elution volume for each of the standards; *V*_t_ is the total column volume calculated as the volume of packed beads; and *V*_0_ is the void volume, which was calculated as 1/3 of the total column volume (Anonymous, 2002).

For immunoprecipitation, 20 μl from each fraction was added to 140 μl 10 mM Tris/HCl (pH 7.4), containing 0.25 M sucrose and 60 μg polyclonal antibody (ApoB, ApoAI and ApoE) or 10 μg mAb [NS3, NS4A, E1 and E2 antibody (CBH-2; from Dr S. Foung, Stanford University, USA)]. After 4 h incubation at 4 °C, 25 μl from 50 % Protein G Sepharose (Gammabind; GE Healthcare) was added and tubes were rotated at 11 r.p.m. for 16 h. Pellets and supernatants were separated by centrifugation at 100 ***g***, at 4 °C for 3 min. Normal rabbit IgG (DakoCytomation) was used as control. HCV RNA in pellet and supernatant was quantified by qRT-PCR.

## RESULTS

### qRT-PCR assays for positive- and negative-strand HCV RNA

**Fig. 1. f1:**
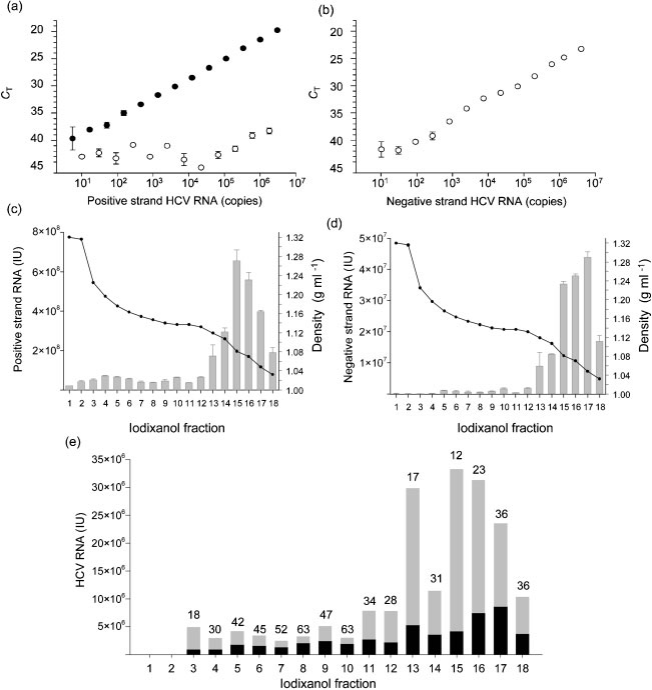
qRT-PCR assays for positive and negative strand HCV RNA from liver. (a, b) *C*_T_ values were measured using a dilution series of either synthetic positive strand HCV RNA (a) or negative strand HCV RNA (b) as a template. One primer set and probe was designed to detect positive strand HCV RNA (•) and another primer set was designed with a tagged primer to detect negative strand HCV RNA (○). Each data point represents the mean±the range of values from two determinations. (c, d) The density distribution of positive (c) and negative (d) strand HCV RNA within iodixanol gradients of HCV-infected liver macerate was determined (•). The concentration of RNA is shown by bar graphs which represent the mean±the range of values from two determinations. (e) Immunoprecipitation of HCV in density gradient fractions with antibody to ApoB. The total HCV positive-strand RNA in the pellets and the supernatants (shaded bars) and the HCV RNA associated with ApoB (solid bars) was calculated as a percentage (given above the bars) for each fraction.

Whilst only positive-strand HCV RNA is packaged into virus particles, both positive- and negative-strand HCV RNA are present in virus replication complexes and serve as intermediates in virus replication. With the aim of determining the specificity and sensitivity of our assays for positive and negative strand HCV RNA, dilution series with synthetic HCV RNA were prepared. The dilutions were used in qRT-PCR assays and the number of PCR cycles (*C*_T_) taken to reach the fluorescence threshold was measured (Fig. 1). A linear correlation was observed between *C*_T_ and positive strand HCV RNA (Fig. 1a[Fig f1]). The sensitivity of this assay was 100 copies per tube and linearity was observed over the entire range of dilutions. In contrast, when the assay for negative strand was used with positive strand HCV RNA template (Fig. 1a[Fig f1]), *C*_T_ values were between 38 and 45, which was considered a negative result. Thus, positive strand RNA did not give a detectable signal in the assay for negative strand. When the assay for negative strand HCV RNA was used with negative strand template, a linear correlation was observed between *C*_T_ values and copy number (Fig. 1b[Fig f1]). These results confirmed the specificity of our negative strand assay, although the sensitivity was lower than that of the positive strand assay, at 1000 copies per tube.

The density distribution of positive- and negative-strand HCV RNA from liver macerate was analysed on iodixanol gradients. Five fractions with low density (fractions 14–18) contained 74 % of the positive-strand RNA, with a peak in fraction 15, which had a density of 1.08 g ml^−1^ (Fig. 1c[Fig f1]). These fractions also contained 90 % of the negative strand, with the peak at 1.05 g ml^−1^ in fraction 17 (Fig. 1d[Fig f1]). The ratio of positive- to negative-strand RNA was calculated for each fraction and found to be lowest in fraction 17 (6 : 1), suggesting that this fraction contains membranes where virus assembly takes place. In iodixanol fraction 15, the ratio was 12 : 1, similar to that seen in cell culture (Quinkert *et al.*, 2005). The percentage of HCV associated with ApoB in each fraction was determined by immunoprecipitation (Fig. 1e[Fig f1]). This analysis showed that the association with ApoB was variable across the density gradient, with a tendency for less association to occur in the low-density fractions, where most of the HCV RNA was detected. In fraction 15, 12 % of HCV RNA was associated with ApoB.

### Distribution of lipid subclasses and proteins within iodixanol density gradients

**Fig. 2. f2:**
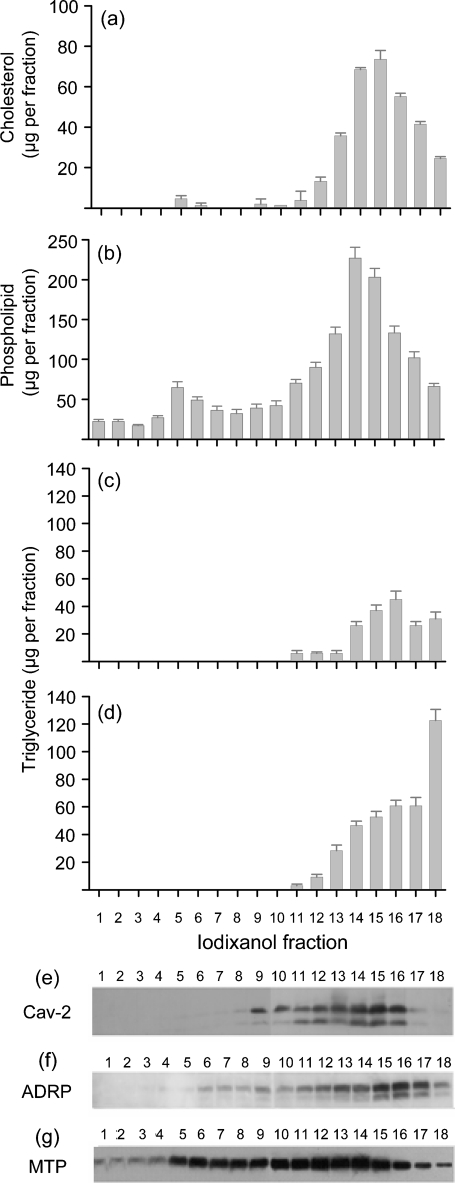
Distribution of lipids (a–d) and proteins (e–g) involved in lipid metabolism within iodixanol density gradient fractions 1–18. Cholesterol (a), phospholipid (b) and triglyceride (d) were measured in each density gradient fraction from liver S6b. Triglyceride was also measured within density gradient fractions from an HCV-negative liver (c). The density distribution of Cav-2 (e), ADRP (f) and MTP (g) in liver S6b was determined by Western blotting.

The distribution of lipids from HCV-infected liver S6b within self-forming iodixanol gradients was compared with the lipid profile for an HCV-negative liver (Fig. 2). Cholesterol and phospholipid levels peaked in fractions 14 and 15 (Fig. 2a, b[Fig f2]) and the distribution of these lipids was similar in the HCV-negative liver (data not shown). Most cholesterol (90 %) was in the form of free cholesterol. In contrast, the largest amount of triglyceride was found in fraction 18 (Fig. 2d[Fig f2]). The HCV-negative liver had significantly less triglyceride in fraction 18 (*P*≤0.005) and all other iodixanol fractions contained less triglyceride than the S6b liver (Fig. 2c[Fig f2]). The distribution within the gradient of proteins involved in lipid metabolism was determined by Western blotting. Caveolin-2 (Cav-2), a marker for lipid rafts (Shi *et al.*, 2003), showed a peak in fractions 14–16 (Fig. 2l[Fig f2]). Adipocyte differentiation-related protein (ADRP), a marker for lipid droplets (Shavinskaya *et al.*, 2007; Targett-Adams *et al.*, 2003), showed a peak in fractions 16–17 (Fig. 2f[Fig f2]). Microsomal transfer protein (MTP), which transfers triglyceride onto the nascent ApoB-100 molecule in the lumen of the endoplasmic reticulum (ER) (Rustaeus *et al.*, 1999), was widely distributed within the gradient, with a peak in fraction 14 (Fig. 2g[Fig f2]).

The distribution of host and viral proteins in iodixanol fractions was also analysed using densitometry of immunostained Western blots (Table 1). Host lipoprotein ApoB showed a peak in fraction 17 (density 1.05 g ml^−1^). Smaller amounts of ApoB were detected in fractions 16 and 18 (densities 1.07 and ≤1.03 g ml^−1^, respectively). Similar distribution of ApoB was observed in an HCV-negative liver (data not shown) and the distribution overlaps with the distribution of triglyceride (Fig. 2c, d[Fig f2]). Host ApoE was widely distributed within the gradient with a peak in fraction 15 (density 1.08 g ml^−1^). ApoAI and ADRP, other host proteins involved in lipid metabolism, as well as lipid raft protein Cav-2 and HCV structural proteins core, E1 and E2 also showed peak intensity in fractions 13 to 17. Although NS3 was widely distributed in the gradient, this viral protein also showed a peak in fraction 15, the same fraction that contained the peak of cholesterol. In combination, these observations suggest that the membranes involved in HCV replication fractionate with a density around 1.08 g ml^−1^ in iodixanol gradients. A third group of proteins was found at a higher density, around 1.12 g ml^−1^, and included ER protein calreticulin and NS4A. Albumin, an abundant cytosolic protein in hepatocytes, showed a peak at the bottom of the gradient, in fractions 1 and 2.

### Analysis of low-density iodixanol fractions by EM and detergent treatment

**Fig. 3. f3:**
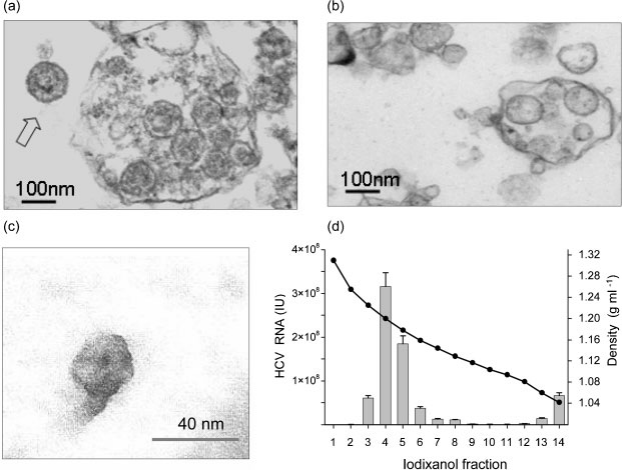
Thin-section EM of fraction 15 from iodixanol gradients of HCV-positive liver (a) or a control HCV-negative liver (b). The fraction from the HCV-positive liver had a density of 1.08 g ml^−1^. The arrow points to an isolated particle which shows internal structure in the form of a pentagon; the diameter of this is 100 nm and the width of the membrane surrounding the particle is 10 nm. (c) Iodixanol fraction 15 was treated with 3 % NP-40 and analysed by negative-staining EM. The density (•) and concentration (bars) of HCV RNA following this treatment (d) indicate that it shifted the density of HCV RNA to 1.2 g ml^−1^, corresponding to the density of the HCV nucleocapsid (Ishida *et al.*, 2001).

Membranes with densities of 1.08 g ml^−1^ from HCV-positive and HCV-negative livers were analysed by thin section EM (Fig. 3a, b[Fig f3]). Bags of particles surrounded by a lipid bilayer, some of which contained an internal structure in the form of pentagons or hexagons, were observed (Fig. 3a[Fig f3]). Similar structures were rarely observed in the HCV-negative liver and lacked the regularly shaped internal structures that were characteristic of the structures in infected liver (Fig. 3b[Fig f3]). Treatment with 3 % NP-40 removed lipids and generated structures resembling the HCV nucleocapsid (Fig. 3c[Fig f3]). The density of these particles was 1.20 g ml^−1^, determined by iodixanol gradient centrifugation, with a recovery of 70 % (Fig. 3d[Fig f3]). This density corresponds to that of the HCV nucleocapsid (Ishida *et al.*, 2001).

### Separation of HCV RNA-containing membranes by gel filtration

**Fig. 4. f4:**
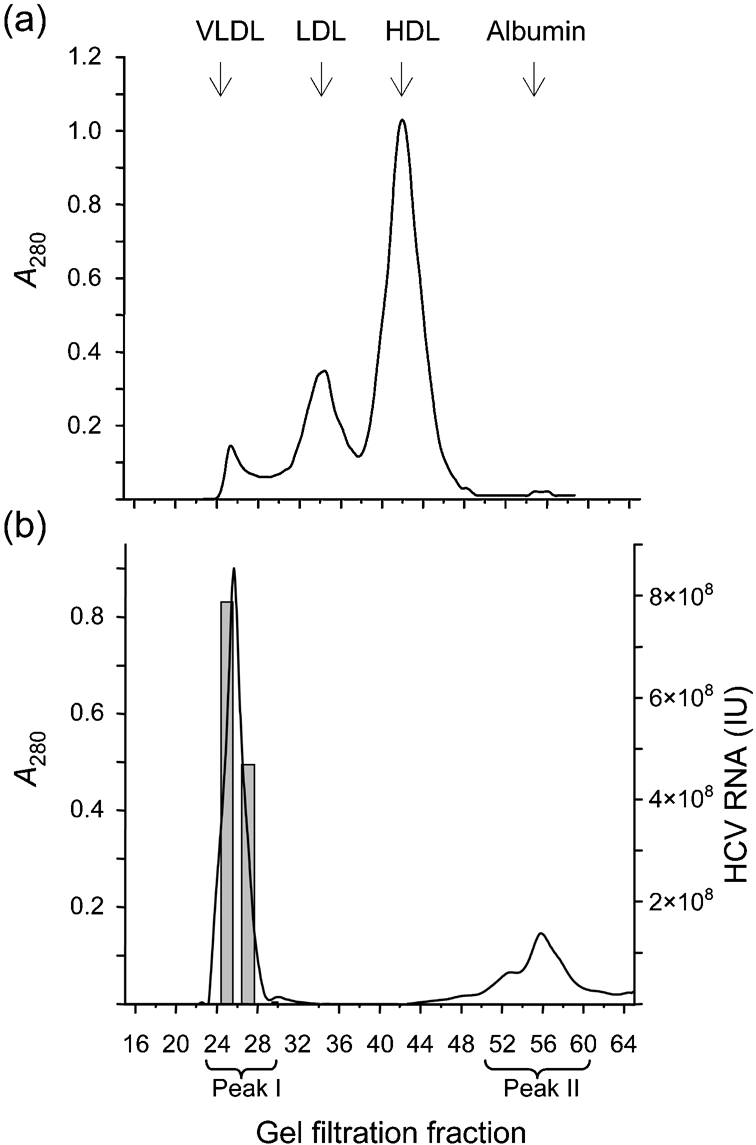
Separation of HCV RNA-containing particles from liver S6b by gel filtration on Superose 6. (a) The column was calibrated using purified lipoproteins and albumin. Peaks corresponding to VLDL, LDL, HDL and albumin are labelled. (b) Gel-filtration of liver macerate first purified on iodixanol gradients, with density below 1.12 g ml^−1^. Bars show the titre of positive strand HCV RNA in fractions collected from the column. The line shows the *A*_280_ of proteins eluting from the gel filtration column. The analysis was repeated four times with similar results.

Gel filtration on Superose 6 was used to separate HCV RNA-containing membranes by size. The column was calibrated with purified VLDL, LDL and HDL, which eluted in three distinct peaks (Fig. 4a[Fig f4]). The molecular mass of VLDL is between 5×10^6^ Da for small VLDL (VLDL2) and 10^7^ Da for large VLDL (VLDL1). LDL has a molecular mass of 2.4×10^6^ Da and that of HDL is between 200 and 400 kDa (Pownall *et al.*, 1999). The molecular mass of VLDL1 exceeds the exclusion limit of Superose 6 (5×10^6^ Da) and it therefore elutes with the void volume. Liver homogenate was first purified on iodixanol gradients and fractions with HCV RNA-containing membranes of less than 1.12 g ml^−1^ (peaks 13–18) were applied to the gel filtration column (Fig. 4b[Fig f4]). All HCV RNA eluted in peak I, at the same position as VLDL. This shows that HCV in liver macerate is within membranes or particles which are greater than or equal to the size of VLDL. Peak II eluted at the same position as human serum albumin and did not contain viral RNA.

### Identification of host and viral proteins in gel-filtration peaks

**Fig. 5. f5:**
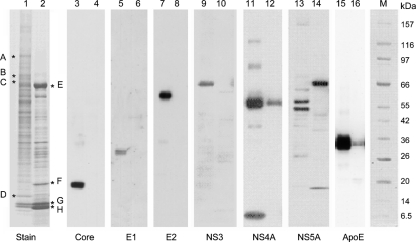
Identification of host and viral proteins after separation by gel filtration. Proteins from gel filtration peaks I and II (Fig. 4b[Fig f4]) were analysed by SDS-PAGE (lanes 1 and 2) and Western blotting (lanes 3 to 16). HCV core protein (20 kDa, lane 3) as well as HCV glycoprotein E1 (31 kDa, lane 5) and glycoprotein E2 (62 kDa, lane 7) were detected in peak I but not in peak II (lanes 4, 6 and 8). HCV proteins NS3 (62 kDa, lane 9) and NS4A (7 kDa, lane 11) were also detected in peak I, but not in peak II (lanes 10 and 12). The antibody to HCV NS5A recognized two bands of 55 and 58 kDa in peak I (lane 13). These bands were not present in peak II (lane 14), but cross-reactivity to albumin at 66 kDa was observed. Host lipoprotein ApoE with molecular mass 36 kDa was recognized in peaks I and II (lanes 15 and 16). Proteins A–H in lanes 1 and 2 were excised and identified by MALDI–TOF (Table 2). Lane M, molecular mass marker.

Proteins in peaks I and II from Superose gel filtration of liver macerate were analysed by SDS-PAGE (Fig. 5). Both peaks contained numerous different proteins (Fig. 5, lanes 1 and 2). Eight of these were excised and analysed by mass spectrometry (Table 2). This analysis identified four proteins in peak I, all ER proteins, and four proteins in peak II, all cytosolic proteins. Proteins from peaks I and II were also analysed by Western blotting, using a panel of monoclonal and polyclonal antibodies to HCV structural and NS proteins. Antibodies to HCV structural proteins core, E1 and E2 identified protein bands with expected molecular masses of 20, 31 and 62 kDa, respectively (Fig. 5, lanes 3, 5 and 7). These structural proteins were detected in peak I but not in peak II. The mAb to NS3 recognized a 69 kDa band in peak I (Fig. 5, lane 9). The human mAb to NS4A recognized a 7 kDa band in peak I (Fig. 5, lane 11), which is the expected mass for NS4A (Waris *et al.*, 2004); this band was absent from peak II. A 56 kDa protein band was also detected in peaks I and II. This band was present in the absence of primary antibody and was identified as human IgG heavy chain. The polyclonal antibody to NS5A recognized two bands of 55 and 58 kDa in peak I but not in peak II (Fig. 5, lanes 13 and 14). These bands were also detected using a monoclonal NS5A antibody from Maine Biotechnology, Inc. (Tami Pilot-Matias, personal communication). These proteins are similar in size to the 56 and 58 kDa basally phosphorylated or hyper-phosphorylated forms of NS5A that were previously observed using *in vitro* expression systems (Neddermann *et al.*, 1999), suggesting that phosphorylated variants of NS5A are present in the HCV-infected human liver. A 66 kDa protein which co-migrated with human albumin was detected by the NS5A antibody (Fig. 5, lane 14), possibly as a result of cross-reactivity. The largest amount of ApoB and ApoE was found in peak I, although a small proportion of ApoE was observed in peak II (Fig. 5, lane 16).

### Separation of HCV RNA-containing membranes on Toyopearl

**Fig. 6. f6:**
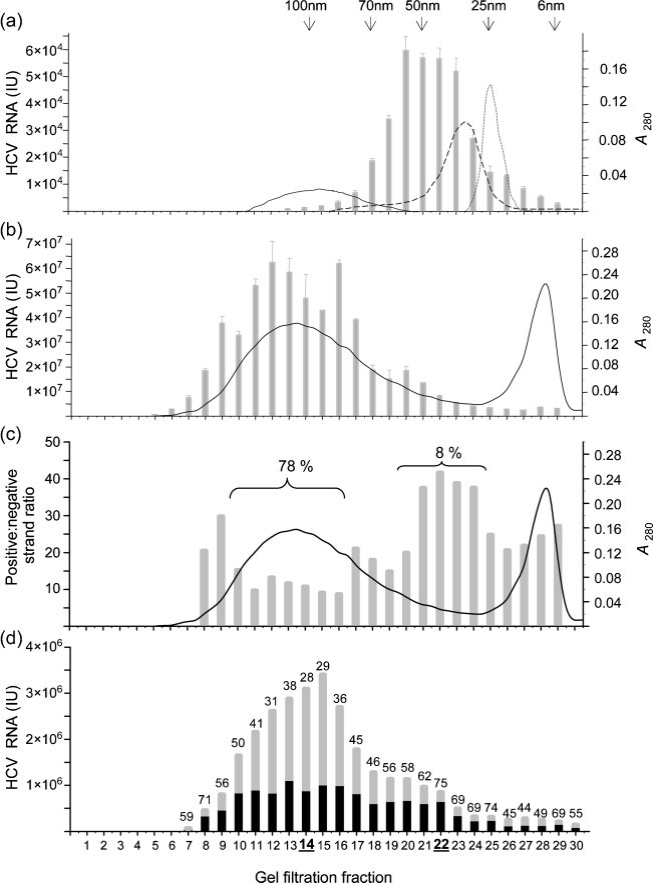
Analysis of serum and liver macerate from patient S6 by gel filtration on Toyopearl. The gel filtration column was calibrated with latex beads of different sizes (indicated by arrows) as well as with purified chylomicrons, VLDL and LDL. The elution profiles of these proteins are shown as solid, dashed and dotted lines, respectively, in (a). (a) Separation of HCV RNA in serum by gel filtration. Bars show the mean±sd HCV RNA in each fraction. (b) Elution profile of total protein (line). Bars show mean±sd (from three determinations) of HCV RNA from liver macerate with density below 1.12 g ml^−1^. (c) The ratio of HCV positive strand RNA and HCV negative strand RNA for each fraction. The proportion of HCV RNA within fractions enclosed by brackets is shown as a percentage. (d) Immunoprecipitation of HCV with antibody to ApoB. The total HCV RNA in the pellets and the supernatants (shaded bars) and the HCV RNA associated with ApoB (solid bars) was calculated as a percentage (given above the bars) for each fraction.

The high exclusion limit of Toyopearl S75 enabled separation of vesicles with larger diameters, up to 40×10^6^ Da, compared with separation on Superose, and allowed the resolution of purified chylomicrons, VLDL and LDL (Fig. 6a[Fig f6]). When serum from patient S6 was analysed on Toyopearl, HCV RNA eluted with VLDL1 (Fig. 6a[Fig f6]). This showed that HCV in serum is similar in size to VLDL1, with a diameter in the region of 60 nm, and supports our previous determination of the HCV diameter by sedimentation analysis (Nielsen *et al.*, 2006). HCV RNA from liver eluted in a broad peak from the Toyopearl column (Fig. 6b[Fig f6]), indicating that most HCV in liver was within membranes with diameter ≥100 nm. However, some HCV in the liver eluted at the same position as HCV from serum, between fractions 20 and 24. Toyopearl fractions 10 to 16 from liver S6b had a low positive-/negative-strand ratio (minimum 6 : 1), which suggested that these fractions contain more of the replication complexes for HCV (Fig. 6c[Fig f6]). Toyopearl fractions 20 to 24 had a high positive : negative strand ratio (maximum 45 : 1). These fractions had high relative association with ApoB (up to 75 %, Fig. 6d[Fig f6]) and HCV RNA was associated with ApoE, ApoAI and HCV E2 (Supplementary Table S1, available in JGV Online). In contrast, viral RNA in Toyopearl-separated fractions 10 to 16 was immunoprecipitated with the NS3 antibody but not with antibodies to HCV E1 or E2 (Supplementary Table S1).

## DISCUSSION

Blood samples and the transplant liver from patient S6 were obtained early, during the acute phase of HCV reinfection. These circumstances provided a unique opportunity to study HCV infection *in vivo* and to characterize the native virus. These samples are also unique as the patient's common variable immunodeficiency enabled us to study virus particles without the complications of host antibodies binding to the virus.

The HCV replication complex contains both positive and negative strand HCV RNA and, in *in vitro* systems, the ratio of the two falls to between 6 : 1 and 12 : 1 (Quinkert *et al.*, 2005). Reports of the positive- to negative-strand ratio in unfractionated HCV-RNA-positive livers vary widely, from 1 : 1 to 1100 : 1 (Chang *et al.*, 2003; Komurian-Pradel *et al.*, 2004; Negro *et al.*, 1998). This variability may reflect the difficulties of precise and specific RNA quantification that occur with low copy numbers of viral RNA in such a complex milieu.

HCV replication complexes have been found associated with lipid rafts and lipid droplets (Aizaki *et al.*, 2004; El-Hage & Luo, 2003; Quinkert *et al.*, 2005). The membranes in iodixanol fraction 15 (positive : negative strand ratio 12 : 1) peak at a density of 1.08 g ml^−1^ and they were enriched in HCV structural and NS proteins, Cav-2, ADRP, phospholipid and cholesterol . This density is somewhat lower than the bulk of the ER proteins, e.g. calreticulin and MTP, which are found at a density of 1.12 g ml^−1^. The low density of membranes containing positive and negative strand HCV RNA suggests that virus replication and assembly occur on structures containing lipids, as previously suggested by Dubuisson *et al.* (2002), Kapadia & Chisari (2005) and Miyanari *et al.* (2007).

The peaks of ApoB and triglyceride were found in iodixanol fractions that had a density below 1.06 g ml^−1^; this density fraction contains Golgi-derived vesicles (Plonne *et al.*, 1999). Phospholipid, cholesterol and ApoE peaked at slightly higher densities, corresponding with the peaks of positive and negative strand HCV RNA. Other members of the family *Flaviviridae*, such as Dengue virus and Kunjin virus, have been found to replicate in vesicle packets that are virus-induced membrane structures in the perinuclear region (Uchil & Satchidanandam, 2003; Westaway *et al.*, 1997). The assembly of VLDL also occurs in the ER and Golgi, with clusters of lipoproteins accumulating in the Golgi lumen (Tran *et al.*, 2002). Our results suggest that the assembly of HCV is linked to the assembly of VLDL, as has recently been suggested by Gastaminza *et al.* (2008). However, ApoB was observed in fractions of slightly lower density than the peak of positive and negative strand HCV RNA, suggesting that viral replication occurs on membranes with higher density than the Golgi clusters filled with VLDL particles.

We found that NS3, NS4A and NS5A co-fractionated with HCV RNA and host VLDL on a Superose 6 gel-filtration column and that those viral proteins had similar molecular masses in human liver as they did in recombinant expression systems (Diaz *et al.*, 2006; Kalamvoki *et al.*, 2006; Nomura-Takigawa *et al.*, 2006). The detection of 55 and 58 kDa bands with anti-NS5A suggests that the variably phosphorylated forms of this protein observed *in vitro* may also exist *in vivo*.

Gel filtration of HCV from serum in a matrix with a large exclusion limit, Toyopearl, led to separation of viral RNA from the main peak of VLDL. The diameter of HCV lipo–viro particles from plasma was determined by gel filtration on Toyopearl and found to be similar to the 55 nm determined previously by sedimentation analysis (Gastaminza *et al.*, 2006; Nielsen *et al.*, 2006) and by immuno-EM (Wakita *et al.*, 2005). This diameter is similar to VLDL1 or chylomicron remnants, but is smaller than chylomicrons. Diaz *et al.* (2006) observed that up to 50 % of HCV in plasma is associated with chylomicrons or chylomicron remnants. Toyopearl gel filtration separated differently sized fractions containing viral RNA. Most HCV RNA was in membranes >100 nm in diameter that were associated with NS3, but 8 % eluted with lower diameter and high positive-/negative-strand ratio. This viral RNA was associated with ApoB, ApoE, HCV E1 and E2, and the diameter corresponded to HCV from serum.

In summary, the combination of iodixanol density gradients and gel filtration has the ability to separate HCV RNA-containing membranes by density and size. This analysis suggests that the association of HCV with lipoproteins occurs in the human liver. The association between HCV and lipoprotein has been observed by others (Gastaminza *et al.*, 2008; Huang *et al.*, 2007; Moradpour *et al.*, 2007; Thomssen *et al.*, 1993; Yao & Ye, 2008) and our techniques show that there is the potential to separate hepatitis C virions from the intracellular membranes where virus assembly occurs.

## Supplementary Material

[Supplementary Table]

## Figures and Tables

**Table 1. t1:** Density distribution of host and HCV proteins from liver macerate Albumin was most abundant in fractions 1 and 2. All of the other proteins listed were most abundant between fractions 13 and 18.

**Protein**	**Subcellular location**	Fraction and density (g ml^−1^)*							
		**7**	**9**	**11**	**13**	**15**	**16**	**17**	**18**
		**1.15**	**1.14**	**1.13**	**1.12**	**1.08**	**1.07**	**1.05**	**≤1.03**
ApoB	Lipoprotein	0	0	0	0	1	22	52	28
ApoE	Lipoprotein	13	18	25	18	34	17	11	6
Apo AI	Lipoprotein	23	27	22	34	41	35	13	7
ADRP	Lipid droplet	8	9	12	20	30	34	24	13
Cav-2	Lipid rafts	0	5	8	18	32	23	2	1
Calreticulin	ER lumen	5	5	42	84	41	26	6	0
MTP	ER lumen	25	37	54	61	47	29	13	6
Albumin	Cytosol	24	21	9	1	0	0	0	0
HCV core	Structural	12	1	4	19	58	15	0	0
HCV E1	Structural	1	1	5	4	10	28	11	6
HCV E2	Structural	4	5	5	67	118	36	6	0
HCV NS3	Non-structural	17	23	22	26	72	25	24	11
HCV NS4A	Non-structural	0	0	8	45	8	7	0	0

*Proteins in iodixanol gradient fractions were analysed by Western blotting and semi-quantified by densitometry.

**Table 2. t2:** Identification of host proteins in peaks from gel filtration of human liver macerate The protein bands were excised from SDS-PAGE gels and the letters assigned to each protein band are shown in Fig. 5, lanes 1 and 2.

**Human protein**	**Cellular location**	**Protein band**	**Observed mass (kDa)**	**Database mass (kDa)**	Probability score*	**Sequence coverage†**
Endoplasmin	ER	A	100	92.4	209	38
NADPH cytochromeP450 reductase	ER	B	75	76.5	88	27
Annexin VI	ER	C	68	75.8	168	40
Glutathione *S* transferase	ER	D	16	17.5	86	74
Serum albumin	Cytosol	E	66	65.6	276	62
Superoxide dismutase (Cu-Zn)	Cytosol	F	19	15.9	94	79
Haemoglobin *β* chain	Cytosol	G	15	15.7	127	65
Haemoglobin *α* chain	Cytosol	H	14	14.9	92	49

*Probability score greater than 64 indicates a significant match with a protein from the NCBI database (*P*<0.05). †Percentage of protein sequence covered by peptides identified in mass spectrometry analysis.
