# Supplementary material for: Characterization of hepatitis C RNA-containing particles from human liver by density and size
Source: J Gen Virol. 2008 Oct;89(Pt 10):2507–17. doi: 10.1099/vir.0.2008/000083-0 (PMC2557069; doi:10.1099/vir.0.2008/000083-0)
Supplement: [Supplementary Table] [file supp_89_10_2507__index.html]

 Characterization of hepatitis C RNA-containing particles from human liver by density and size -- Nielsen et al. 89 (10): 2507 Data Supplement - Supplementary Table -- Journal of General Virology

## Supplementary Table S1

### Characterization of hepatitis C RNA-containing particles from human liver by density and size, by S. U. Nielsen, M. F. Bassendine, C. Martin, D. Lowther, P. J. Purcell, B. J. King, D. Neely and G. L. Toms

*Journal of General Virology* vol. **89**, part 10, pp. 2507 - 2517

**Supplementary Table S1.** Immunoprecipitation of HCV from gel filtration fractions 14 and 22 with antibodies to HCV proteins and host lipoproteins. [PDF] (12 kb)

  
  
