# Supplementary material for: Characterization of hepatitis C RNA-containing particles from human liver by density and size
Source: J Gen Virol. 2008 Oct;89(Pt 10):2507–17. doi: 10.1099/vir.0.2008/000083-0 (PMC2557069; doi:10.1099/vir.0.2008/000083-0)
Supplement: [Supplementary Table] [file supp_89_10_2507__1.pdf]

Supplementary Table S1. Immunoprecipitation of HCV from gel filtration fractions 14 and 22 with antibodies to HCV proteins and host lipoproteins.

	Percentage of HCV RNA precipitated from fractions 14 and 22						
	Control	NS3	NS4A	ApoE	ApoAI	E1	E2
Fraction **14**	1	42	17	12	6	3	3
Fraction **22**	7	17	10	51	34	16	32

Nielsen, S. U., Bassendine, M. F., Martin, C., Lowther, D., Purcell, P. J., King, B. J., Neely, D. and Toms, G. L. (2008). Characterization of hepatitis C RNA containing particles from human liver by density and size. *J Gen Virol* **89**, 2507–2517.
